# Isolation and identification of probiotic *Bacillus subtilis* AJQ03 from the intestinal tract of *Anguilla japonica* (Japanese eel)

**DOI:** 10.3389/fmicb.2024.1446299

**Published:** 2024-10-30

**Authors:** Xi Wang, Yuxin Yao, Hui Ge, Jiaonan Zhang, Jiaolin Zhang, Qingpi Yan

**Affiliations:** ^1^Fisheries College, Jimei University, Xiamen, Fujian, China; ^2^Fisheries Research Institute of Fujian, Xiamen, Fujian, China; ^3^Key Laboratory of Special Aquatic Feed for Fujian, Fujian Tianma Technology Company Limited, Fuzhou, Fujian, China

**Keywords:** *Anguilla japonica*, *Bacillus subtilis*, adhesion inhibition, intestinal colonization, probiotics

## Abstract

In recent years, the use of fish-derived probiotics in aquaculture has become more widespread. However, research on *Anguilla japonica*-derived probiotics is still limited. To evaluate the potential of probiotics for disease control in eel aquaculture, isolates were obtained from the intestinal tract of healthy *Anguilla japonica*. These isolates were assessed for their adhesion properties, inhibition of pathogen adhesion, and hydrolytic enzyme production. Morphological characteristics and 16S rRNA sequence analysis were used for identification. Results showed that the AJQ03 strain adhered to the intestinal mucus and inhibited common pathogenic bacteria through adhesion inhibition, and further produced amylase, lipase, protease, and cellulase. Based on morphological characteristics and 16S rRNA sequencing, AJQ03 was identified as *Bacillus subtilis*. The strain demonstrated tolerance to various extreme conditions, as well as survival in simulated gastrointestinal fluids and superior growth in intestinal fluid compared to Luria-Bertani (LB) broth. *In vitro* safety tests showed that AJQ03 was not resistant to 32 antibiotics and exhibited γ hemolysis on blood plate. *In vivo* safety tests demonstrated a 100% survival rate for the fish, with stable organ indices, reduced bacterial loads in the liver and spleen, and complete bacterial clearance by day 7 without residue. Intestinal bacterial load results confirmed effective colonization by strain AJQ03. Analysis of the impact of AJQ03 on the gut microbiota of *A. japonica* revealed a significant increase in the relative abundance of *Bacillus* at the genus level, corroborating the colonization efficiency of AJQ03. Additionally, the relative abundances of *Klebsiella*, *Pseudomonas*, and *Aeromonas* were significantly lower compared to the controls, indicating that strain AJQ03 effectively reduced harmful bacteria and improved gut microbiota composition. This study confirms that *B. subtilis* AJQ03, isolated from the intestine of *A. japonica*, can serve as a probiotic candidate in *A. japonica* aquaculture.

## 1 Introduction

*Anguilla japonica*, commonly known as the Japanese eel, is a typical anadromous fish species that grows in rivers and lakes, but migrates to the deep sea to reproduce upon reaching sexual maturity (Ye et al., 2023). Known for its delicious meat, high nutritional content, and significant commercial value (Okamoto et al., 2009), it is a major aquaculture species in many East Asian countries (Takeuchi et al., 2019). However, rapid expansion of aquaculture operations, driven by the unilateral pursuit of economic benefits at the expense of appropriate scientific management, has led to various issues such as water quality deterioration, density stress, and nutritional imbalance, resulting in increased risk of bacterial disease outbreaks, impacting the healthy development of the eel industry (Yuan et al., 2021).

Disease epidemics in aquaculture often result from the misuse of antibiotics, leading to drug residues, drug-resistant strains, and environmental pollution and food safety concerns, posing threats to both the aquaculture industry and human health (Lieke et al., 2020). Probiotics have emerged as an effective alternative to antibiotics for disease treatment and prevention. The aquacultural application of probiotics began nearly 40 years ago when *Bacillus toyoi* spores were fed to amberjack as a feed additive, resulting in enhanced growth of the fish (Kozasa, 1986). As the use of probiotics in fish aquaculture has increased, they have significantly improved the industry; however, for successful application, probiotics must be screened based on various selection criteria (Merrifield et al., 2010; Wang et al., 2019), including safety, adaptability, functionality, and convenience, and their efficacy must be investigated both *in vitro* and *in vivo* (Nayak et al., 2023).

Common forage probiotics in the aquaculture industry include *Bacillus*, *Lactobacillus*, *Clostridium*, and *Saccharomyces* (Balcázar et al., 2006). *Bacillus* species are particularly notable for their antibacterial and antibiofilm activities, rapid growth, low nutrient requirements, and anaerobic tolerance (Nayak, 2021). These species often secrete hydrolytic enzymes such as extracellular protease, amylase, cellulase, and lipase, aiding aquatic animals in utilizing nutrients in their feed (Zokaeifar et al., 2012; Yu et al., 2009). *Bacillus* species also meet the Food and Agriculture Organization (FAO) and World Health Organization (WHO) criteria for probiotics, making them ideal probiotic candidates (Binda et al., 2020)*. Lactobacillus* species inhibit harmful bacteria through acid production or bacteriocins and play an important role in maintaining intestinal flora balance in fish (Gatesoupe, 2008). *Clostridium butyricum* promotes the proliferation of beneficial flora, inhibits harmful bacteria, repairs damaged intestinal mucosa, reduces inflammation, and enhances host immunity (Zhang et al., 2020).

Despite the predominance of terrestrial animal-origin probiotics in aquaculture, due to extensive studies on terrestrial gut microorganisms and the stability of probiotic traits (Jang et al., 2022), recent research suggests that host-derived probiotics offer greater health benefits to the host, including evasion of host defenses, adaptation to the host gut environment, immunomodulation, and nutrient conversion (Banerjee et al., 2017; Jang et al., 2021; Hien et al., 2020). However, there is limited research on host-derived probiotics for *A. japonica*. To address this, we screened and identified host-derived probiotics with beneficial properties for *A. japonica*. Notably, a strain of *B. subtilis* (AJQ03) was identified from 21 bacterial strains isolated and purified from the intestinal tract of healthy *A. japonica*. This strain was selected for its strong extracellular enzyme production, superior adhesion ability, and effectiveness in preventing pathogenic bacterial adhesion. Overall, this study provides candidate strains for the development and application of probiotics in *A. japonica* aquaculture.

## 2 Materials and methods

### 2.1 Ethics statement

All animal experiments were approved by the Animal Ethics Committee of Jimei University (permit number JMULAC2011-58) and were carried out in compliance with the National Institutes of Health’s Guide for the Care and Use of Laboratory Animals.

### 2.2 Isolation of *Bacillus* from *Anguilla japonica* intestine

Healthy fish (500–600 g) were anesthetized with MS-222 (100 ppm, West Gene, China) for 5 min. The intestines were then extracted and rinsed three times with phosphate-buffered saline (PBS, Biosharp, China) to remove the contents. The intestines were divided into foregut, midgut, and hindgut sections, followed by the addition of an appropriate amount of PBS at a 1:9 mass ratio. These sections were then homogenized for 60 s at 45 Hz using a tissue grinder (Jingxin Technology, Shanghai, China). The isolation method was modified from previously described protocols (Yan et al., 2018). In brief, each homogenate was heated in a water bath at 60°C for 1 h. After dilution, the homogenate was coated on *Bacillus* megatherium agar plates (Huangkai Microbial Technology, Guangdong, China) and cultured at 28°C for 48 h. Single colonies were selected for morphological observation and gram staining to screen for gram-positive with morphological characteristics consistent with *Bacillus*. Bacteria were streaked three times for purification, and then stored on agar medium.

### 2.3 *In vitro* adhesion capacity

#### 2.3.1 Bacterial culture and *in vitro* mucus preparation

Strains were cultured in LB broth at 28°C for 24 h, and adjusted to an optical density at 600 nm (OD_600_ nm) = 0.3 ± 0.01. Mucins were prepared according to previously described methods (Yang et al., 2023). The gastrointestinal tract of healthy *A. japonica* was harvested and washed with PBS and the inner surface of the intestine scraped to obtain the mucus-protein mixture. The mucus was centrifuged at 4°C and 4,000 rpm for 30 min, and the supernatant was collected and filtered sequentially through 1.0 μm and 0.45 μm pore size filters to remove bacteria. The protein concentration of the mucus was adjusted to 1 mg/mL according to a quantitative kit (TransGen Biotech, Beijing, China), and the mucus was dispensed and stored at −20°C.

#### 2.3.2 *In vitro* adhesion test

An *in vitro* adhesion assay was conducted according to established protocols (Xin et al., 2022). Briefly, 20 μL of the prepared mucus was evenly spread on a glass slide (22 mm × 22 mm, Biosharp, China). Subsequent to the mucus drying, 200 μL of 4% methanol solution was added dropwise for fixing the mucus for 2 h. 200 μL of bacterial solution was spread evenly over the mucus area of the slides and incubated in a humid environment at 37°C for 2 h, rinsed with PBS, and air dried. 200 μL of 4% methanol was added to the slides to fix the adherent cells for 30 min to achieve immobilization and then stained with 200 μL of 1% crystal violet staining solution for 2 min, rinsed with PBS, and air dried. Adhered bacteria in 20 randomly selected fields were counted under a microscope (×200) (Leica DM4000 B LED, Leica, Germany). Three independent biological replicates were performed per group.

### 2.4 Inhibitory effects of AJQ03 on pathogenic adhesion

Five pathogenic strains susceptible to *A. japonica* (*Pseudomonas plecoglossicida*, *Edwardella trade*, *Vibrio harveyi*, *Vibrio anguillarum*, and *Aeromonas hydrophila*) were selected for the study. The three primary types of adhesion inhibition (competition, substitution, and rejection) were investigated.

The experiment followed previous research (Xia et al., 2024), with some modifications. Strains were cultured in LB broth at 28°C for 24 h, and adjusted to an optical density at 600 nm (OD_600_ nm) = 0.3 ± 0.01. In the competitive adhesion inhibition test, 100 μL of pathogenic bacteria solution and 100 μL of probiotic solution were added dropwise to mucus-fixed slides and incubated at 37°C for 2 h, then rinsed with PBS and air dried. In the substitution adhesion inhibition test, mucus-fixed slides were incubated with 100 μL of pathogenic bacteria solution at 37°C for 1 h, rinsed with PBS, incubated with 100 μL of probiotic solution at 37°C for 1 h, rinsed with PBS, and air dried. In the rejection adhesion inhibition test, mucus-fixed slides were incubated with 100 μL of probiotic solution for 1 h at 37°C, rinsed with PBS, then incubated with 100 μL of pathogenic bacteria solution at 37°C for 1 h, rinsed with PBS, and air dried. After incubation, the slides were fixed with 200 μL of 4% methanol and subjected to Gram staining. Adhered bacteria in 20 randomly selected fields were counted under a microscope (×200). Three independent biological replicates were performed per group.


$$\begin{array}{l} \mathrm{Adhesion}\;\mathrm{inhibition}\;\mathrm{rate}\;\left(\%\right)=\\ \frac{\left(\mathrm{control}\;\mathrm{group}\;\mathrm{adhesion}-\mathrm{experimental}\;\mathrm{group}\;\mathrm{adhesion}\right)}{\mathrm{control}\;\mathrm{group}\;\mathrm{adhesion}}\;\times 100\% \end{array}$$

### 2.5 Extracellular enzyme production

The activities of digestive enzymes, including amylase, protease, cellulase, and lipase (Zhang et al., 2021), were evaluated using extracellular enzyme production plate assays of potential probiotics, as described in previous studies (Muñoz-Atienza et al., 2014), with some modifications.

Protease and lipase production were determined using agar plates supplemented with 2% skimmed milk and 1% Tween 80, respectively. The cultured bacterial solution (10 μL, OD_600_ = 0.3 ± 0.01) was inoculated into sterile perforated 6 mm wells and incubated at 28°C for 24 h, with clearing around the wells indicating protein hydrolysis and a white calcium ring indicating fat hydrolysis. Amylase production was determined using agar plates supplemented with 1% starch. The cultured bacterial solution (10 μL) was inoculated into sterile perforated 6 mm wells and incubated at 28°C for 24 h. After incubation, the plates were treated with 1% Lugol’s iodine solution, with clearing around the wells indicating starch hydrolysis. Cellulase production was assessed using agar plates supplemented with 2% carboxymethyl cellulose agar. The cultured bacterial solution (10 μL) was inoculated into sterile perforated 6 mm wells and incubated at 28°C for 24 h. After incubation, the plates were treated with 1% Congo red dye, with clearing around the wells indicating cellulose hydrolysis. All experiments were conducted at least three times.

### 2.6 Molecular identification

Total bacterial DNA was extracted using a genomic DNA rapid extraction kit (Vazyme Biotech, Nanjing, China). The 16S rRNA of strain AJQ03 was amplified using universal primers 27F (5′-AGAGTTTGATCCTGGCTCAG-3′) and 1492R (5′-GGTTACCTTGTTACGACTT-3′). The results were analyzed using 1% agarose gel electrophoresis. The polymerase chain reaction (PCR) product of the target band was then sent to Sangon Biotech Co., Ltd. (China) for sequencing. The obtained 16S rRNA sequence was subjected to sequence alignment analysis using BLAST against the NCBI database, and sequences with high similarity to the potential probiotic were downloaded. A phylogenetic tree was constructed using the neighbor-joining (N-J) method with MEGA v11.0.

### 2.7 Tolerance determination

#### 2.7.1 Tolerance test of pH, bile salt, and NaCl

The pH tolerance test was performed following previous research (Nayak et al., 2023), with some modifications. In brief, the cultured bacterial solution was inoculated into LB broth with different pH values (2.0, 4.0, 6.0, 7.0, 8.0, 10.0, and 12.0) at a 10% inoculation volume (OD_600_ = 0.6 ± 0.01) and incubated at 28°C and 220 rpm for 24 h. Absorbance at OD_600_ was measured using pH 7.0 as the control.

The bile salt tolerance test was performed following previous research (Nayak et al., 2023), with some modifications. In brief, the cultured bacterial solution was inoculated into LB broth with different bile salts (Biosharp, China) concentrations (0, 0.15, 0.30, 0.45, and 0.60%) at a 10% inoculation volume (OD_600_ = 0.6 ± 0.01) and cultured at 28°C and 220 rpm for 24 h. Absorbance at OD_600_ was measured using a bile salt concentration of 0% as the control.

The NaCl tolerance test was performed following previous research (Nayak et al., 2023), with some modifications. In brief, the cultured bacterial solution was inoculated into LB broth with different NaCl (Macklin, China) concentrations (0.5, 1.5, 2.5, 3.5, 4.5, 5.5, and 6.5%) at a 10% inoculation volume (OD_600_ = 0.6 ± 0.01) and cultured at 28°C and 220 rpm for 4 h. Absorbance at OD_600_ was measured using a NaCl concentration of 0.5% as the control.

#### 2.7.2 Tolerance in simulated gastrointestinal fluids

The tolerance test in simulated gastrointestinal fluids was modified from previously described methods (Saba et al., 2023). Given the fluctuating pH of gastric fluid, pH was adjusted to 2.0, 3.0, and 4.0, respectively. To prepare the simulated gastric fluid, 1 mg/mL pepsin (Yuanye, China) was added to the LB broth and the pH was adjusted to 2, 3, and 4 using 10% HCl. The solution was mixed and filtered through a 0.22 μm membrane to eliminate bacteria, and the he cultured bacterial solution was inoculated into the simulated gastric fluid with different pH values at a 10% inoculation volume (OD_600_ = 1.0 ± 0.01) and cultured at 28°C and 220 rpm for 4 h. Absorbance at OD_600_ was measured using simulated gastric fluid treatment at 0 min as the control. For the simulated artificial intestinal fluid preparation, 1.36 g of KH_2_PO_4_ (Macklin, China) was added to 200 mL of LB broth, the pH was adjusted to 6.8, and then added to 0.42 g of trypsin (Yuanye, China), mixed, and filtered through a 0.22 μm membrane to remove bacteria. The cultured bacterial solution was inoculated into simulated intestinal fluid at a 10% inoculation volume (OD_600_ = 1.0 ± 0.01) and cultured at 28°C and 220 rpm for 6 h. Absorbance at OD_600_ was measured using incubation in LB broth for 6 h as the control.

### 2.8 *In vitro* safety tests

#### 2.8.1 Antibiotic sensitivity test

The drug sensitivity test was performed using the Kirby-Bauer disk diffusion method (Li et al., 2020) with 32 commonly used drug susceptible disks (Hangzhou Microbiology Reagent Co., Ltd., China). The cultured bacterial solution (100 μL, OD_600_ = 0.3 ± 0.01) was coated on LB agar plates, with the antibiotic disks then placed on the surface. The plates were incubated at 28°C for 12 h, and the diameter of the transparent inhibition zone around the disks was measured. Antimicrobial susceptibility results were interpreted according to the Clinical and Laboratory Standards Institute (CLSI) guidelines. Strains were classified as sensitive (S), intermediate (I), or resistant (R) (Cockerill et al., 2012).

#### 2.8.2 Hemolysis test

Hemolytic activity was assessed as described previously (Eaton et al., 2001). In brief, 10 μL of bacterial solution (OD_600_ = 0.3 ± 0.01) was added to wells in agar plates containing 1% defibrinated sheep blood (Guangdong Huankai Microbial Technology Co., Ltd., China). The negative control consisted of 10 μL of LB broth, while the positive control consisted of 10 μL of Triton X-100 (Beyotime, Shanghai, China), incubated at 28°C for 24 h. Colony appearances were observed, and hemolysis was classified into alpha, beta, and gamma types based on the color around the wells (Maragkoudakis et al., 2006).

### 2.9 *In vivo* safety test and colonization

#### 2.9.1 Experimental management

Healthy *A. japonica* (body length 15.0 ± 1.0 cm, weight 56.56 ± 11.85 g) were purchased from the Kang Liang Aquatic Farm (Guangdong, China) and acclimated at 27 ± 1°C for 1 week under specific pathogen-free laboratory conditions. The potential probiotics for infection were cultured in LB broth at 28°C for 20 h to the late logarithmic phase, then centrifuged and resuspended in PBS.

For the survival assay, 180 fish were randomly divided into four groups, each consisting of three tanks. Group A1 fish each received a 0.1-mL intraperitoneal injection of fresh bacterial solution (1 × 10^8^ CFU/mL), while the group B1 control fish each received the same volume of PBS buffer. In the feeding experiment, the bacterial solution was mixed with feed at a rate of 0.2 mL of bacterial solution (1 × 10^8^ CFU/mL) per fish per serving of feed by forced feeding (intragastric) with a syringe. The fish in group A2 were provided with a daily feed containing a freshly prepared bacterial solution, whereas the control fish in group B2 were fed an equal amount of feed mixed with PBS buffer. The fish in both groups were fed continuously with the bacterial feed for 1 week and then switched to normal feed for 1 week. Fish condition and mortality were recorded twice daily, with detailed records of the time, number, and signs of any clinical or pathological changes or deaths during the study. All deceased fish were necropsied to determine the cause of death.

#### 2.9.2 Biosafety evaluation and enteric colonization effects

The injection groups were sampled for the first 7 days, while the feeding groups were sampled on days 1, 7, and 14. At each sampling point, three fish (fasted for 24 h before sampling) were randomly collected from each tank and quickly anesthetized with MS-222. Fish body weight was measured, blood was collected from the dorsal aorta, and liver, spleen, and intestines were isolated with sterile scissors, immediately frozen in liquid nitrogen, and stored at −80°C. Organ indices are important in experiments such as safety evaluations as they reflect the development of organs, assess the potential toxicity of substances and their effects on the immune system, and provide important reference data for subsequent studies (Rauta et al., 2012). The organ indices were determined as described in previous research (Zhong et al., 2016), where intact livers and spleens were weighed and liver and spleen indices were calculated. Bacterial load determination was modified from previously described methods (Chen et al., 2011). The liver, spleen, and intestines were mixed with PBS buffer at a 1:9 mass ratio and homogenized for 60 s at 45 Hz using a tissue grinder. The homogenized samples and blood were then plated on LB agar medium, with the absence of bacterial growth indicative of probiotic safety.


$$\mathrm{Organ}\;\mathrm{indices}=\left(\mathrm{organ}\;\mathrm{fresh}\;\mathrm{weight}\left(g\right)\right)/\left(\mathrm{fish}\;\mathrm{weight}\left(g\right)\;\right)$$

#### 2.9.3 Enteric colonization effects

Intestinal tissues from every two fish in each feeding group were pooled into one sample, three samples per group. All samples were quickly frozen in liquid nitrogen and stored at −80°C for subsequent DNA extraction. Total DNA was extracted from the intestine using the TruSeqTM DNA Sample Prep Kit (QIAGEN, Hilden, Germany) according to the manufacturer’s instructions. DNA concentration was determined and the V3–V4 regions of the DNA samples that passed the quality test were amplified using universal primers 338F (5′-ACTCCTACGGGAGGCAGCAG-3′) and 806R (5′-GGACTACHVGGGTWTCTAAT-3′). The qualified DNAs were sent to Shanghai Majorbio Bio-pharm Technology Co., Ltd., China for Illumina Miseq sequencing. The single long reads and original libraries were spliced by paired-end sequencing using the FLASH (v.1.2.7) program. Sequence quality was controlled using fastp (v.0.19.6) and sequence noise reduction was performed using DADA2. Statistical analysis was conducted using QIIME2 v2022.2. QIIME2 v2022.2. was employed to assess the composition abundance distribution of each sample at phylum and genus levels, and compared with the Sliva database (v.138) to count the community species composition of each sample. Additionally, Alpha diversity was calculated using Mothur (v.1.30.2), including chao1, ACE, Shannon, and Simpson index (Dan et al., 2020).

### 2.10 Statistical analysis

Data normality was assessed by Kolmogorov–Smirnov test and statistical analyses were conducted using one-way analysis of variance (ANOVA) with GraphPad Prism v9.5. Duncan’s multiple comparison test was used to evaluate differences among groups. A *p*-value of less than 0.05 was considered significant. All experiments were performed in triplicate and results are expressed as mean ± standard deviation (SD).

## 3 Results

### 3.1 *In vitro* adhesion capacity

**Figure 1:**
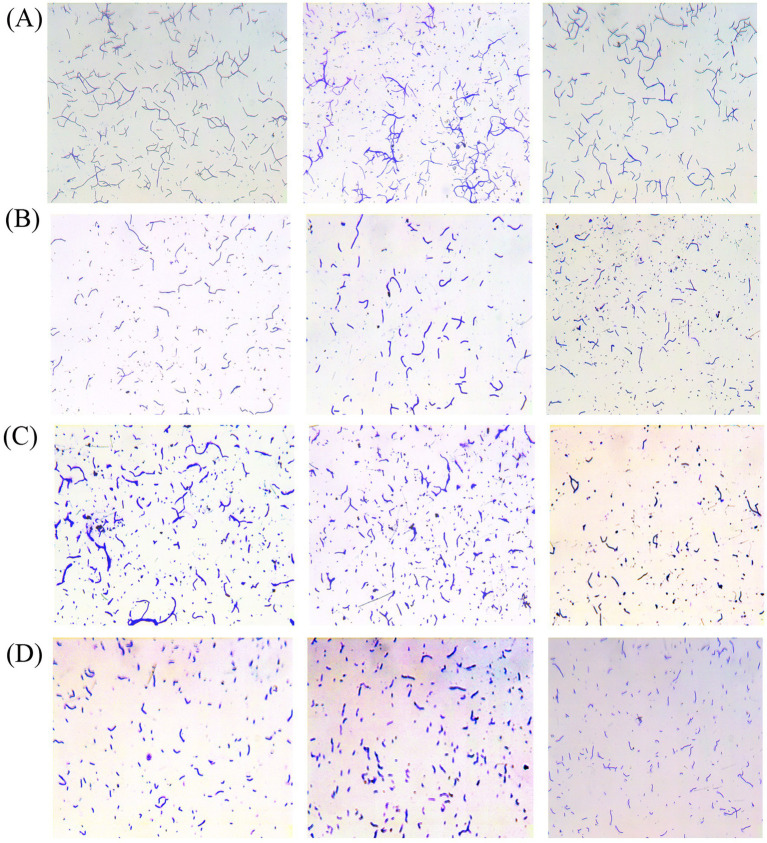
Adhered bacteria observed under the microscope and adhesion ability of strain AJQ05 (*N* = 3) **(A)**, strain AJH03 (*N* = 3) **(B)**, strain AJQ03 (*N* = 3) **(C)**, strain AJZ02 (*N* = 3) **(D)**.

The four eligible strains of bacteria were further analyzed. The adhesion effects of the strains were observed using a microscope, and the adherent bacterial cells were counted in three randomly selected areas under a 200× field of view. The data demonstrated that the mean adhesion of strain AJQ05 was 281 (Figure 1A), while strain AJH03 was 124 (Figure 1B), strain AJQ03 was 244 (Figure 1C), and strain AJZ02 was 144 (Figure 1D). The strains AJQ05 and AJQ03 demonstrated a higher degree of adhesion than the other strains. Accordingly, these two strains were selected for the subsequent screening stage.

### 3.2 *In vitro* adhesion inhibition capacity

The findings indicated that the strains AJQ05 and AJQ03 demonstrated varying degrees of inhibitory efficacy against the five strains of pathogens, with the inhibition expressed as the adhesion inhibition rate.

**Figure 2:**
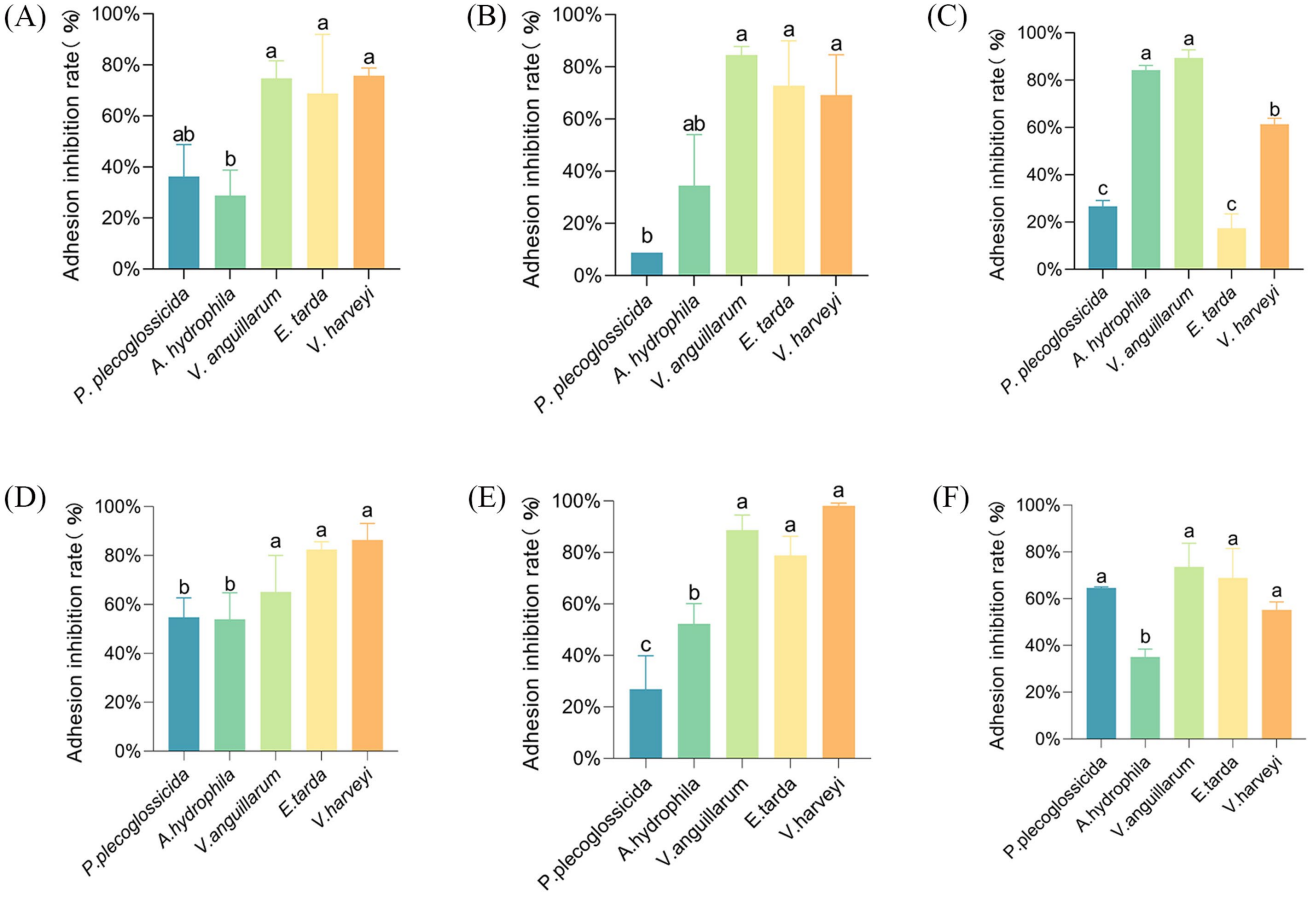
Rate of adhesion inhibition of strain AJQ03 against five pathogenic bacteria by competition (*N* = 3) **(A)**, substitution (*N* = 3) **(B)**, and rejection (*N* = 3) **(C)**; rate of adhesion inhibition of strain AJQ05 against five pathogenic bacteria by competition (*N* = 3) **(D)**, substitution (*N* = 3) **(E)**, and rejection (*N* = 3) **(F)**.

In the competitive adhesion inhibition model, strain AJQ03 showed a higher inhibitory effect on *V. harveyi*, *V. anguillarum*, and *E. tarda*, with inhibition rates of approximately 70% (*p* < 0.05) (Figure 2A). Strain AJQ05 exhibited a notable inhibitory capacity against all five strains of pathogens, with an exceptional adhesion inhibition rate of 86.4% ± 6.73% against *V. harveyi* (*p* < 0.05) (Figure 2D). In the substitution adhesion inhibition model, AJQ03 exhibited higher inhibitory effects on *V. harveyi*, *V. anguillarum*, and *E. tarda*, with the inhibition rate of *V. anguillarum* reaching nearly 85% (*p* < 0.05) (Figure 2B). The strain AJQ05 demonstrated a markedly elevated degree of inhibition of substitution adhesion to *A. hydrophila*, *V. harveyi*, *V. anguillarum*, and *E. tarda* in comparison to *P. plecoglossicida* (*p* < 0.05) (Figure 2E). In the rejection adhesion inhibition model, the strain demonstrated a higher inhibitory effect on *V. anguillarum* and *A. hydrophila*, with the inhibition rate of *V. anguillarum* reaching approximately 90% (*p* < 0.05) (Figure 2C). A significant inhibition of adhesion was observed in strain AJQ05 in comparison with *A. hydrophila* against *P. plecoglossicida*, *E. tarda*, *V. harveyi* and *V. anguillarum* (*p* < 0.05), with the inhibition rate of *V. anguillarum* reaching approximately 80% (Figure 2F). Both strains showed different effects on the ability to inhibit adhesion, so the experiment was continued to the next step.

### 3.3 Extracellular enzyme activity

**Figure 3:**
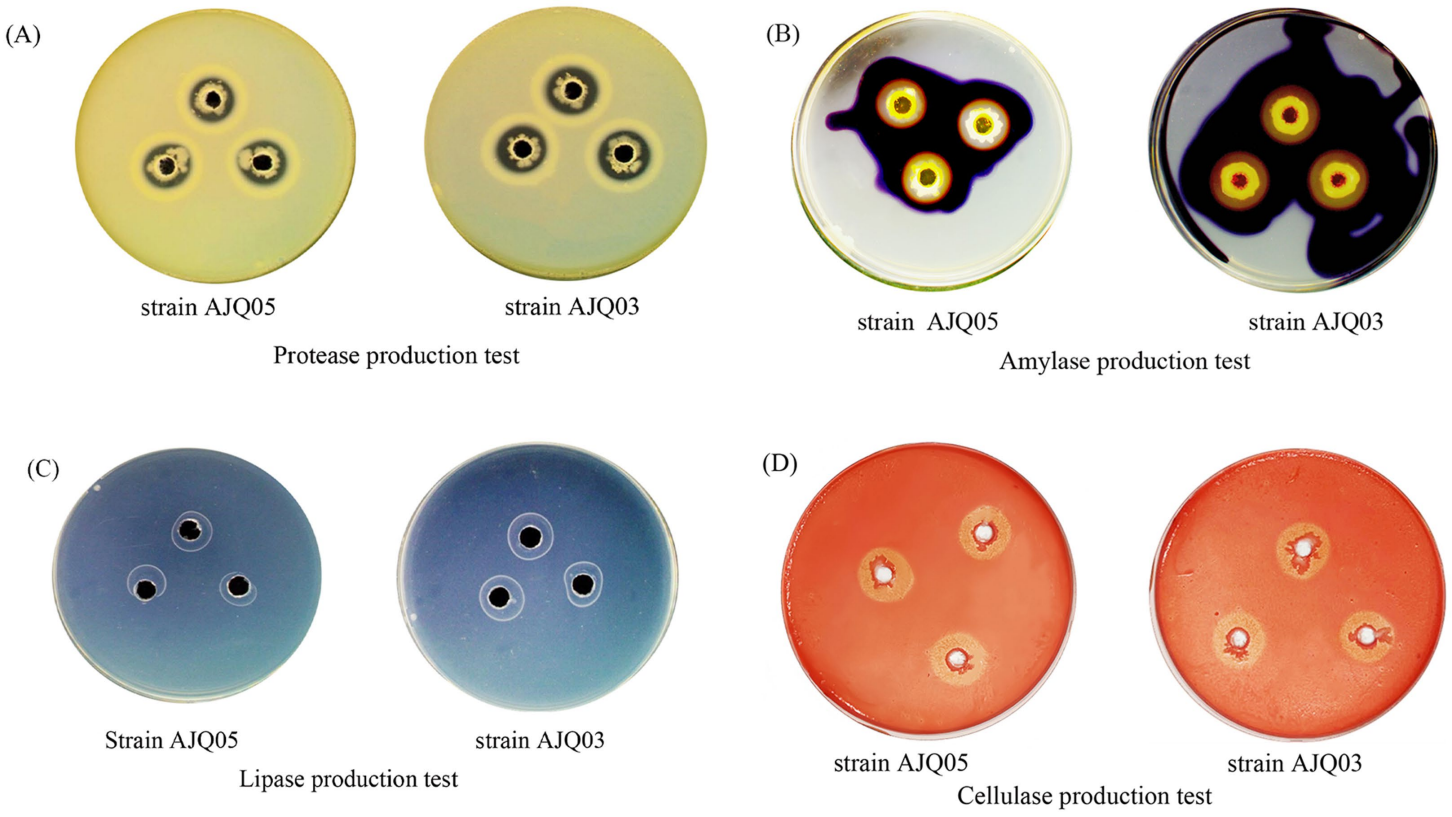
Extracellular enzymatic activities of strains AJQ05 and AJQ03 (*N* = 3).

The results demonstrated that both strains AJQ05 and AJQ03 were capable of secreting amylase, protease, cellulase, and lipase, as evidenced by the hydrolysis circle diameter/colony diameter (H/C) ratios. The H/C values of strain AJQ03 were 2.92 ± 0.19 for protease, 4.18 ± 0.18 for amylase, 2.63 ± 0.09 for lipase and 3.26 ± 0.07 for cellulase. The H/C values of strain AJQ05 were 1.96 ± 0.14 for protease, 2.27 ± 0.11 for amylase, 2.16 ± 0.03 for lipase and 2.12 ± 0.13 for cellulase (Figure 3). The results showed that strain AJQ03 had a strong ability to produce extracellular enzymes, so strain AJQ03 was selected as a potential probiotic candidate.

### 3.4 Identification of potential probiotic isolate

**Figure 4:**
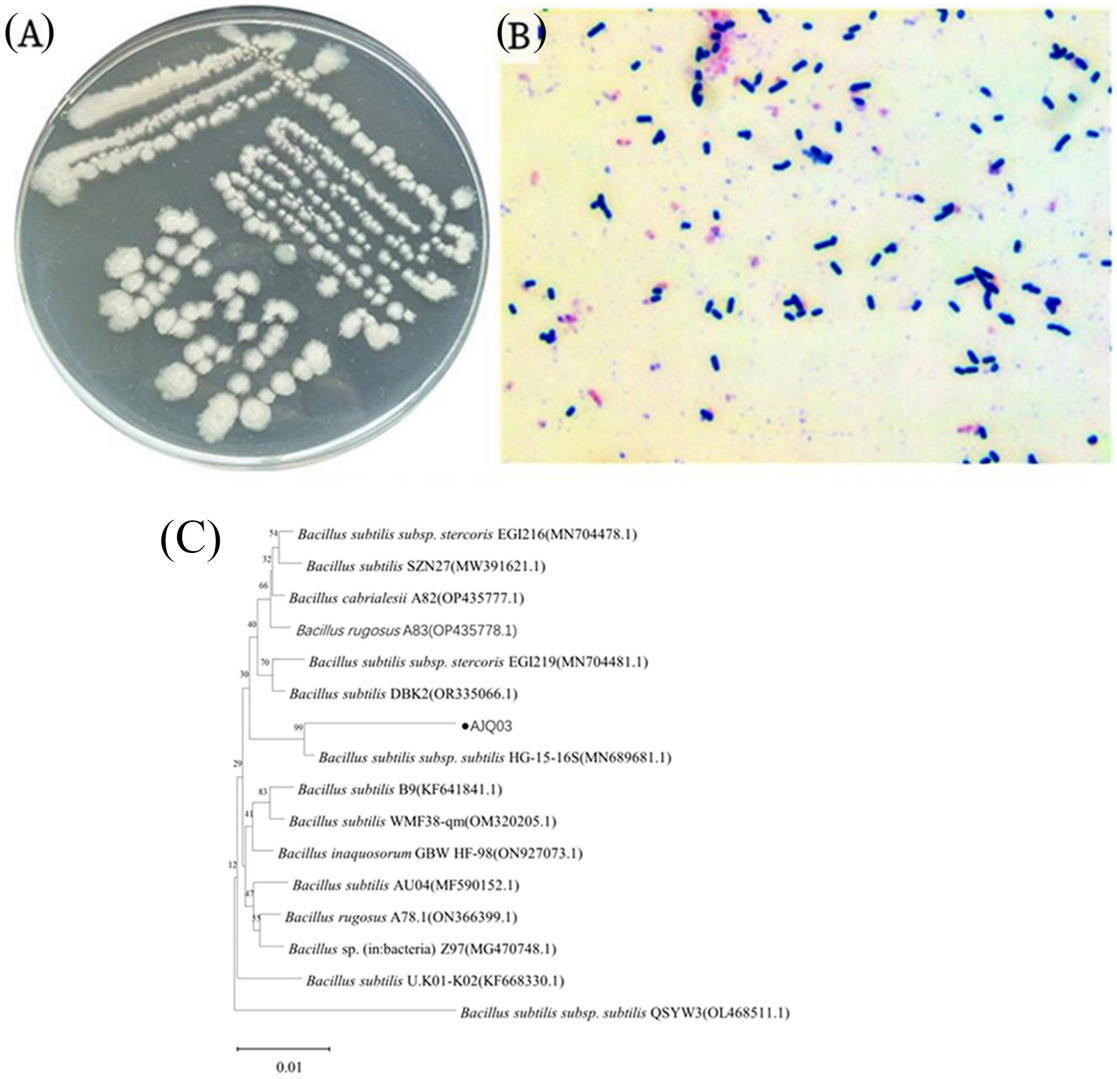
Morphological observations of AJQ03 strain: **(A)** Colonial morphology; **(B)** Gram staining. **(C)** Phylogenetic tree of AJQ03 strain.

Given its high adhesion and enzyme-producing capabilities, strain AJQ03 was selected for identification and further analysis. Notably, the strain exhibited good growth on the LB agar medium, forming transparent, protuberant single colonies with irregular moist edges (Figure 4A). Microscopic examination showed a blue-violet Gram stain, indicating that the bacteria were gram-positive, with straight, rod-shaped bodies not forming chains (Figure 4B). Sequencing revealed a sequence length of 1,141 bp. The 16S rRNA sequence of this isolate showed 99% similarity with *B. subtilis* sequence in the GenBank, and phylogenetic analysis indicated that strain AJQ03 was clustered with *B. subtilis* species, grouping together in the phylogenetic tree (Figure 4C). Consequently, strain AJQ03 was identified as *B. subtilis*. The GenBank accession number of the 16S rRNA sequence of strain AJQ03 is PQ282698.1.

### 3.5 Tolerance tests

#### 3.5.1 Tolerance of strain AJQ03 to pH, bile salts, and NaCl

**Figure 5:**
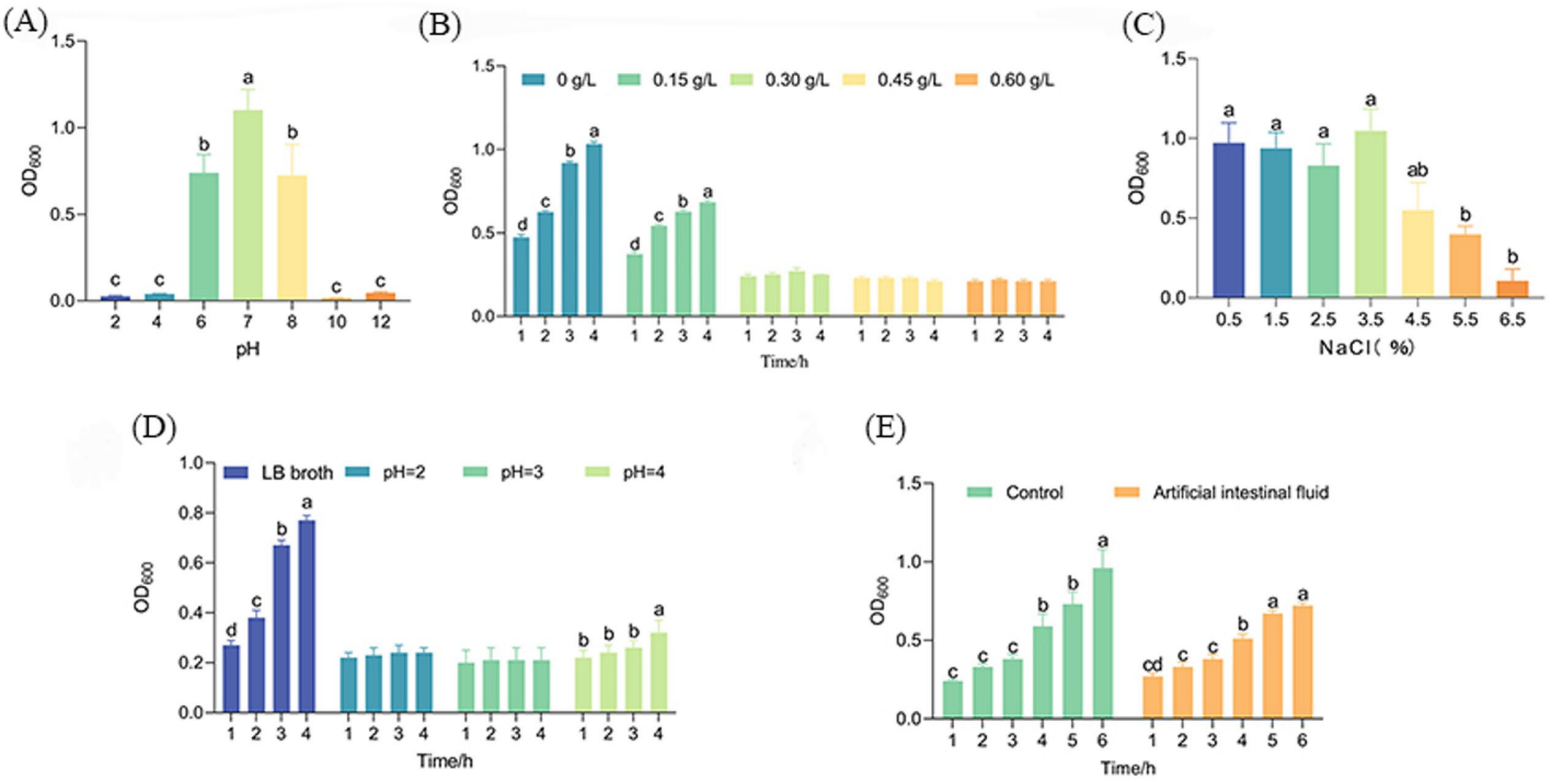
Growth of AJQ03 strain under different **(A)** pH range, **(B)** bile salt concentration, **(C)** NaCl concentration, **(D)** simulated gastric fluids, and **(E)** simulated intestinal fluids (*N* = 3).

The pH resistance test results for strain AJQ03 are presented in Figure 5A. After 24 h in LB broth at different pH levels, the OD_600_ value in the pH 7.0 control group was significantly higher than in other groups, reaching 1.10 ± 0.12 (*p* < 0.05). In acidic environments, at pH 6.0, the OD_600_ was 0.74 ± 0.11. At pH 4.0 and 2.0, the OD_600_ values decreased significantly, but still measured 0.026 at pH 2.0 (*p* < 0.05). In alkaline environments, at pH 8.0, the OD_600_ was 0.72 ± 0.18. At pH 10.0 and 12.0, the OD_600_ values decreased significantly, but still measured 0.05 at pH 12.0 (*p* < 0.05). While the growth capabilities of AJQ03 decreased with increasing acidity and alkalinity, the strain still exhibited some growth, indicating a wide range of acid–base adaptability.

The bile salt tolerance test results for strain AJQ03 are shown in Figure 5B. After 4 h in LB broth supplemented with varying concentrations of pig bile salt, the OD_600_ in the 0 and 0.15% bile salt groups increased significantly (*p* < 0.05), by 0.46 and 0.45, respectively. In the 0.30–0.60% bile salt treatment groups, OD_600_ remained unchanged, indicating that high concentrations of bile salts inhibited AJQ03 strain growth. However, the strain still showed slight growth, demonstrating adaptability to extreme bile salt conditions.

The NaCl tolerance test results for strain AJQ03 are shown in Figure 5C. After 24 h in LB broth supplemented with different concentrations of NaCl, the OD_600_ values in the 1.5–3.5% NaCl treatment groups showed no significant difference from the control (*p* > 0.05). At 4.5–6.5%, the OD_600_ value decreased significantly but remained at 0.11 ± 0.07 under 6.5% NaCl (*p* < 0.05). These findings indicate that while high concentrations of NaCl inhibited strain growth, AJQ03 still exhibited considerable tolerance, demonstrating a wide range of NaCl adaptability.

#### 3.5.2 Tolerance of strain AJQ03 in simulated gastrointestinal fluids

The simulated gastrointestinal fluid results of strain AJQ03 are shown in Figures 5D,E. After 4 h in simulated gastric fluid at different pH levels, the OD_600_ remained unchanged in the pH 2.0–3.0 treatment group but increased significantly in the pH 4.0 treatment group, demonstrating good tolerance to pH fluctuations in the simulated gastric environment (*p* < 0.05, Figure 5D). After 6 h in simulated intestinal fluid, the OD_600_ of the treatment group significantly increased by 0.45 ± 0.02, while the control group in LB broth showed a significant increase of 0.75 ± 0.11 (*p* < 0.05, Figure 5E). These findings suggest that although the simulated intestinal fluid exhibited slight inhibitory effects, AJQ03 still demonstrated good tolerance.

### 3.6 *In vitro* safety tests

#### 3.6.1 Sensitivity of strain AJQ03 to drugs

The antibiotic sensitivity test results for strain AJQ03 against 32 different antibiotics are shown in Table 1. Strain AJQ03 was sensitive to all tested antibiotics, indicating that it did not produce drug resistance and can be considered a safe strain.

**Table 1:** Antibiotic susceptibility of strain AJQ03.

Antibiotics	Drug content/μg	Standard for antibacterial circle diameter/mm			Antibacterial circle diameter/mm	Sensitivity
		Highly susceptible	Intermediate susceptible	Resistant		
Penicillin	1	≥29	27–28	≤26	31.00	S
Oxacillin	1	≥20	15–19	≤14	18.43	I
Ampicillin	10	≥17	14–16	≤13	28.23	S
Carbenicillin	100	≥18	15–17	≤14	35.21	S
Piperacillin	100	>18	17–18	<17	28.25	S
Cefalexin	30	≥20	15–19	≤14	54.43	S
Cefazolin	30	≥18	15–17	≤14	57.40	S
Cefradine	30	≥22	19–21	≤18	59.72	S
Cefuroxime	30	≥23	20–22	≤19	52.51	S
Ceftazidime	30	≥18	15–17	≤14	34.51	S
Ceftriaxone	30	≥21	14–20	≤13	46.22	S
Cefoperazone	75	≥21	16–20	≤15	41.36	S
Amikacin	30	≥27	14–26	≤13	31.05	S
Gentamycin	10	≥15	13–14	≤12	26.94	S
Kanamycin	30	≥18	14–17	≤13	28.72	S
Neomycin	30	≥17	13–16	≤12	28.73	S
Tetracycline	30	≥15	12–14	≤11	23.90	S
Doxycycline	30	≥16	13–15	≤12	34.29	S
Minocycline	30	≥33	30–32	≤29	36.43	S
Erythromycin	15	≥23	14–22	≤13	34.13	S
Medemycin	30	≥35	15–34	≤14	33.23	S
Norfloxacin	30	≥17	13–16	≤12	40.05	S
Ofloxacin	5	≥16	13–15	≤12	43.06	S
Ciprofloxacin	5	≥21	16–20	≤15	42.88	S
Vancomycin	30	≥15	–	<15	25.28	S
Polymyxin B	300	≥40	37–39	≤36	12.83	I
Sulfamethoxazole	23.75	≥16	11–15	≤10	31.09	S
Furazolidone	100	≥42	37–41	≤38	34.17	S
Chloramphenicol	30	≥18	13–17	≤12	33.33	S
Clindamycin	2	≥21	15–20	≤14	29.58	S
Doxycycline	30	≥14	11–13	≤10	28.51	S
Enrofloxacin	10	≥46	43–45	≤42	39.84	S

S, sensitive; I, moderately sensitive; R, drug resistance.

#### 3.6.2 Hemolytic test

**Figure 6:**
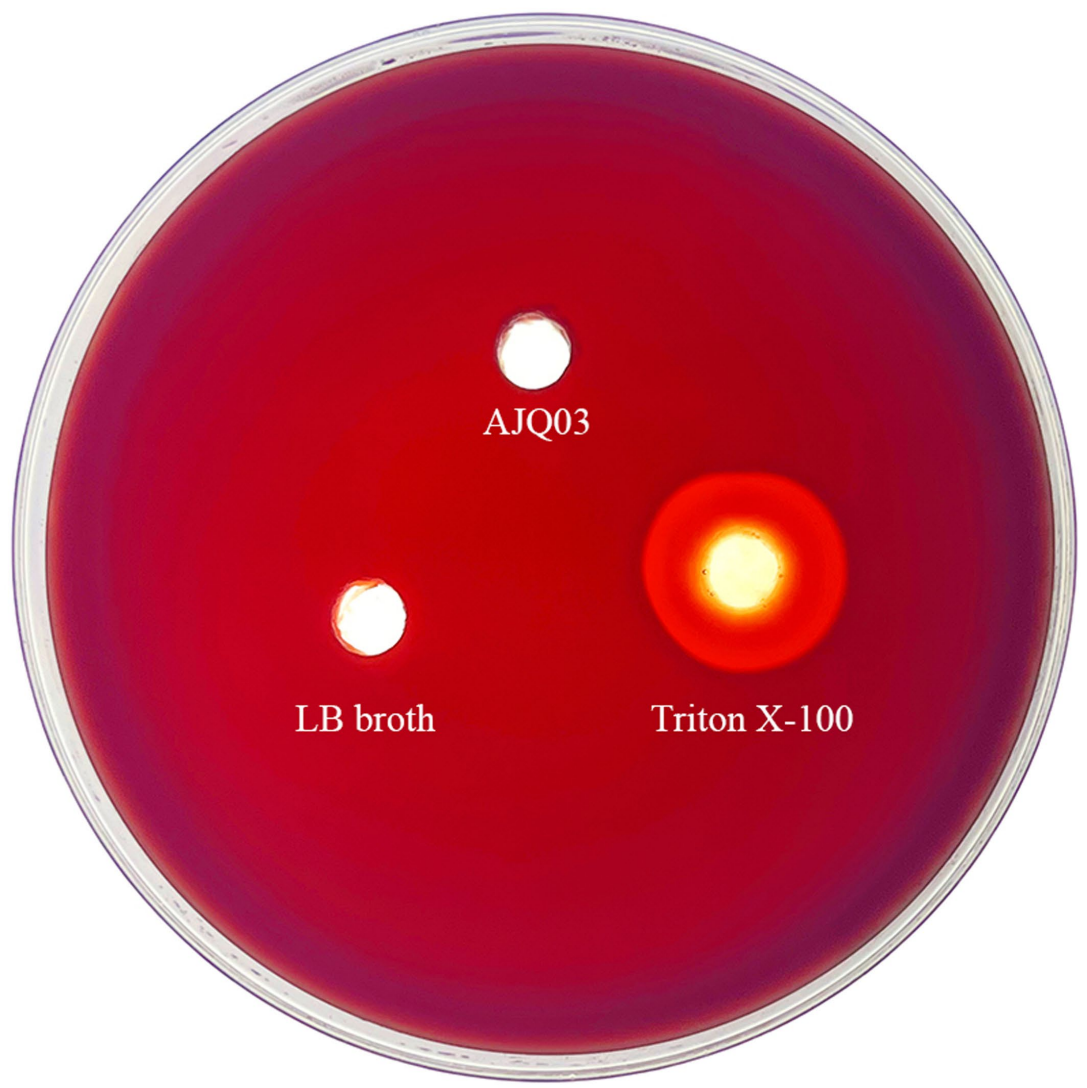
Hemolytic results of strain AJQ03.

Strain AJQ03 exhibited no hemolytic activity (γ-hemolytic) on blood agar, as shown in Figure 6.

### 3.7 *In vivo* safety test and colonization

#### 3.7.1 *In vivo* safety test

**Figure 7:**
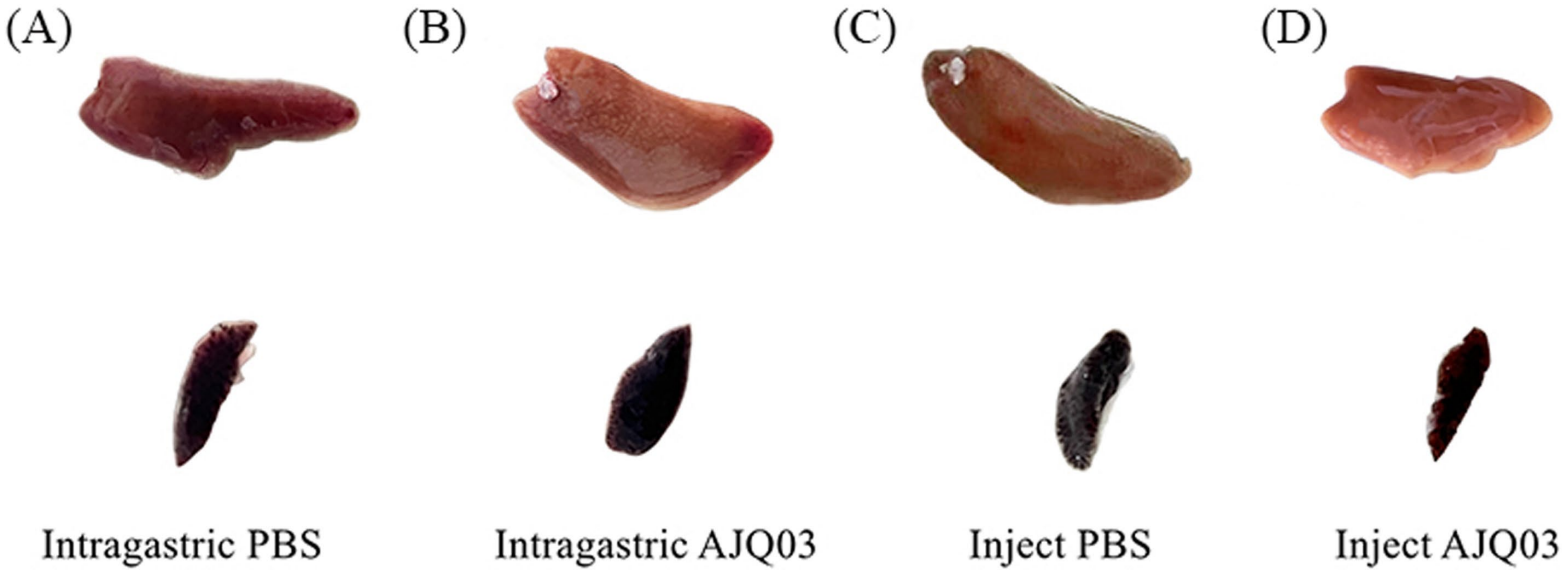
Spleen and liver symptoms after intragastric administration of PBS **(A)** and AJQ03 **(B)** and injection of PBS **(C)** and AJQ03 **(D)** in *A. japonica* (*N* = 3).

**Figure 8:**
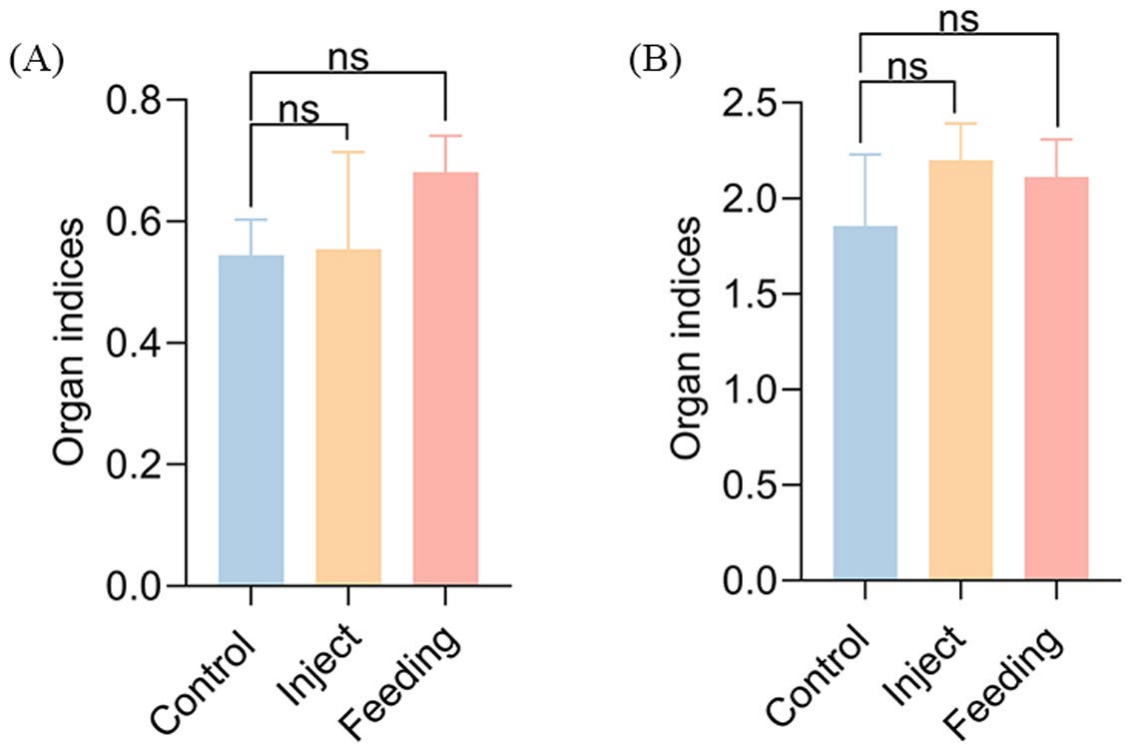
Organ index: **(A)** Spleen index of inject and feeding strain AJQ03, **(B)** Liver index of inject and feeding strain AJQ03.

Strain AJQ03 exhibited no virulence to *A. japonica*, with all groups exhibiting 100% survival at a concentration of 1 × 10^8^ CFU/mL, and no lesions were observed in the livers and spleens of infected fish compared to the control group (Figures 7A–D). Intact livers and spleens were harvested, weighed, and recorded to calculate the liver and spleen indices on day 7, which showed no significant differences compared to the control group (*p* > 0.05, Figure 8).

**Figure 9:**
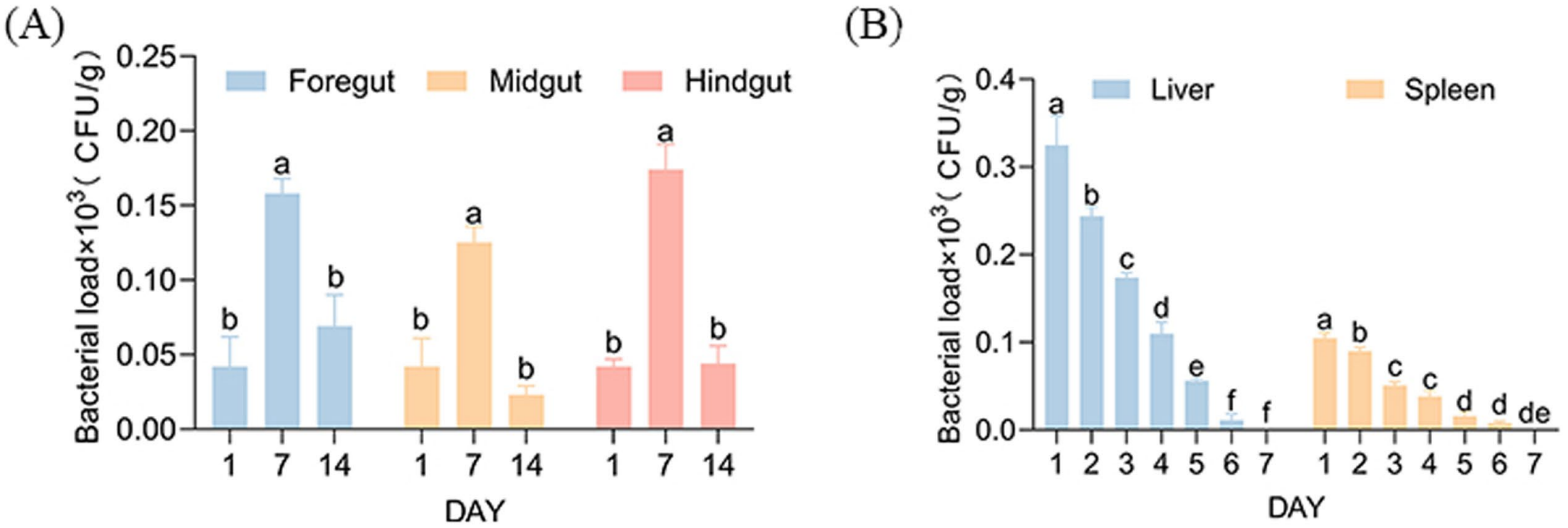
Bacterial loads: **(A)** Intestinal bacterial load after feeding strain AJQ03 and **(B)** Spleen and liver bacterial load after injection of strain AJQ03 (*N* = 3).

In the feeding group, the highest bacterial load was observed in the hindgut on day 7 (1.74 × 10^2^ CFU/g). On day 14, the bacterial load in the foregut reached 0.69 × 10^2^ CFU/g, indicating weak colonization. This indicates that strain AJQ03 might form a colonizing population in the gut (Figure 9A). Accordingly, the composition of the intestinal microbiota was analyzed. In the intraperitoneally injected AJQ03 group, no bacterial strains were detected in the blood, and no bacterial presence was observed in the organs after day 7 (Figure 9B), which further demonstrated that AJQ03 has a high safety profile in the application of *A. japonica*.

#### 3.7.2 Analysis of colonization effects and gut microbiota

**Figure 10:**
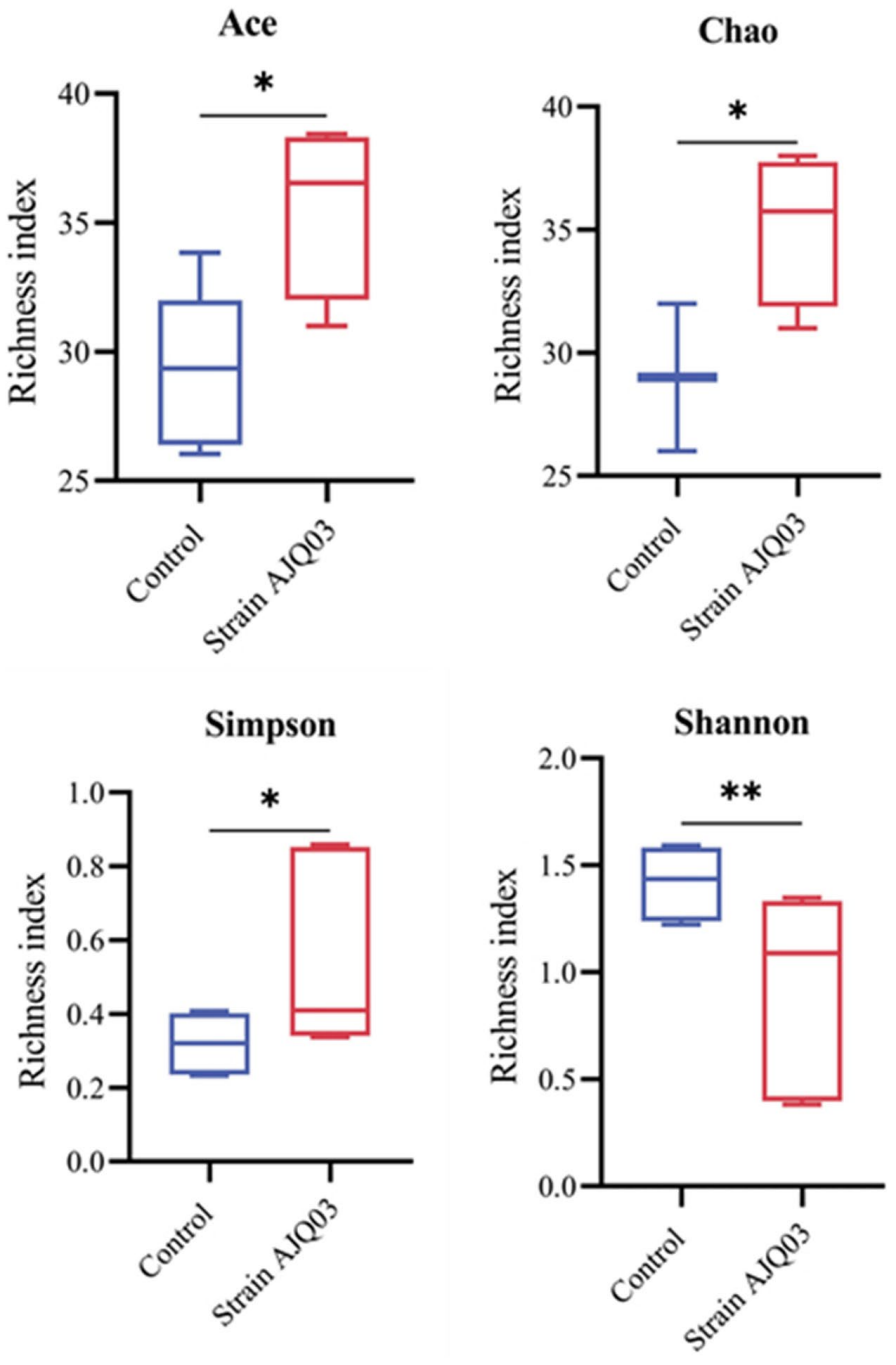
Effects of strain AJQ03 on alpha diversity indices of gut microbes of *A. japonica*. Values are expressed as mean ± SD (*N* = 3). Bars with different “*” represents significant difference (P<0.05), and “**” represents extremely significant difference (P<0.01).

After Illumina sequencing, a total of 689,388 effective sequences were obtained. The DADA2 noise reduction process produced 518,357 sequences across 10 samples, with each sample yielding between 46,089 and 65,303 sequences, resulting in 502 amplicon sequence variants (ASVs), with coverages of all samples exceeding 0.99. Treatment with strain AJQ03 impacted the alpha diversity of the *A. japonica* gut microbiota. As shown in Figure 10 compared to the control group, the Simpson, Chao, and ACE indices in the treatment group increased significantly (*p* < 0.05), while the Shannon index decreased significantly (*p* < 0.01).

**Figure 11:**
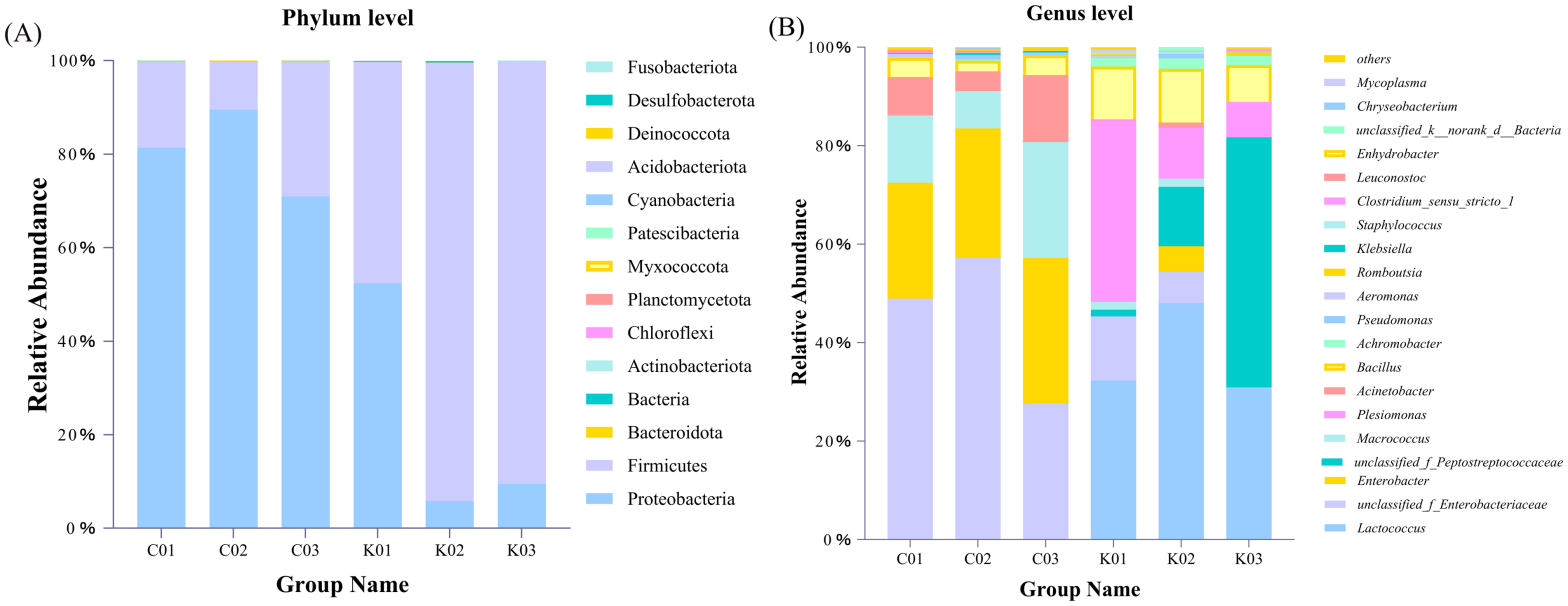
Gut microbial composition of *A. japonica* at the phylum **(A)** and genus **(B)** levels. C01, C02, and C03 indicate control groups. K01, K02, and K03 indicate treatment groups with strain AJQ03.

The gut microbial composition at the phylum and genus levels is shown in Figure 11. At the phylum level, the control group was dominated by Proteobacteria, followed by Firmicutes, Bacteroidota, unknown Bacteria, and Actinobacteriota, with Myxococcota and Deinococcota also detected. In the treatment group, Firmicutes was the most dominant phylum, followed by Proteobacteria, Bacteroidota, unknown Bacteria, and Actinobacteriota, with Chloroflexi, Planctomycetota, Patescibacteria, Acidobacteriota, and Fusobacteriota also detected (Figure 11A).

At the genus level, the top 12 genera in the *A. japonica* intestine were *Lactococcus*, unclassified_f_*Enterobacteriaceae*, *Enterobacter*, unclassified_f_*Peptostreptococcaceae*, *Macrococcus*, *Plesiomonas*, *Bacillus*, *Enhydrobacter*, *Achromobacter*, *Klebsiella*, *Pseudomonas*, and *Aeromonas*. Compared to the control group, the treatment groups showed an increase in the relative abundances of *Lactococcus* and *Bacillus* and a decrease in the relative abundances of *Klebsiella*, *Pseudomonas*, and *Aeromonas* (Figure 11B).

## 4 Discussion

Research has shown that probiotics hold considerable application prospects in aquaculture (Nayak, 2010). Among them, *B. subtilis* is a promising candidate due to its ability to produce endophytic spores, which are highly tolerant, easy to store, and exhibit no toxic side effects (Liu et al., 2009). In this study, strain AJQ03 isolated from the intestinal tract of healthy *A. japonica* demonstrated robust probiotic properties, combined with its morphological characteristics (Liu et al., 2014) and 16S rRNA identification, strain AJQ03 was identified as *B. subtilis*. Probiotics initially use mucin as a substrate for adhesion upon entering the host gut, as they lack the ability to degrade adhesion molecules (Johansson et al., 2013). The capacity of probiotics to adhere to host intestinal mucus is a crucial criterion indicating their adhesive properties. The *in vitro* mucus adhesion model, which accurately represents the adhesion characteristics of probiotics, is widely used in practical applications. For example, Grześkowiak et al. (2011) analyzed the adhesion of four strains of bacteria, including *A. hydrophila*, to mucus from different parts of three fish species using a mucus adhesion model. Xin et al. (2022) evaluated the adhesion characteristics to the intestinal tract and the factors affecting adhesion using intestinal mucus extracted from *Epinephelus fuscoguttatus*. The adhesion and colonization of pathogenic bacteria in the intestinal tract are key factors in the pathogenesis of infectious diseases. Inhibiting the adhesion of these bacteria can reduce cell invasion and morbidity, which is important for maintaining normal immune function, elucidating disease mechanisms, and establishing new infection preventing methods (Zhang et al., 2024). Xiong et al. (2024) suggested that probiotics can reduce disease incidence by decreasing the adhesion capacity and number of pathogenic bacteria in the gut. Generally, models for inhibiting adhesion of pathogenic bacteria by probiotics can be categorized into three types: competition, substitution, and rejection inhibition. Xia et al. (2024) demonstrated that competitive inhibition blockade the adhesion of *A. hydrophila* to the intestinal mucus of loach. The results of this study indicated that strain AJQ03 had varying inhibitory effects on pathogenic bacteria via different types of adhesion inhibition mechanisms.

Extracellular enzymes can complement an animal’s endogenous digestive enzymes, enhancing the breakdown of macromolecular nutrients and improving digestion and absorption (Huyben et al., 2017). Enzymatic activity reflects the somatic efficiency of an organism in digesting nutrients and supporting growth (Ding et al., 2004; Hemarajata et al., 2013). Medison et al. (2023) isolated a *B. licheniformis* strain (YZCUO202005), which produced various extracellular enzymes, including cellulase, protease, amylase, and β-1,3-glucanase. Ramlucken et al. (2020) also identified the beneficial properties of bacteria based on their extracellular enzyme-producing activity. Therefore, the ability to produce extracellular enzymes is a crucial criterion for screening potential probiotics. The *B. subtilis* strain AJQ03 produced a variety of extracellular enzymes with high activity, making it a suitable candidate for probiotic selection.

For probiotics to be effective, they must demonstrate high tolerance and colonization ability in the gastrointestinal tract, as well as withstand extreme conditions in *in vitro* experiments, as demonstrated by their survival in the fish gut (Markowiak et al., 2018; Chaucheyras-Durand et al., 2010). Our study clarified the tolerance range of strain AJQ03, showing that it grows in the pH range of 6–8, in bile salt concentration range of 0 to 0.15 g/L, and in NaCl concentration range of 0.5–5.5%. These findings are consistent with previous studies, even exhibiting a broader tolerance range (Furzikova et al., 1999). As probiotics must pass through the stomach to reach the gut, gastric tolerance is critical (Ramos et al., 2013). The pH of gastric fluid typically fluctuates from 2 to 4. Guo et al. (2016) reported that *B. subtilis* isolated from the intestinal tract of grass carp can tolerate highly acidic environments at pH 2.0. Similarly, in the present study, strain AJQ03 exhibited no significant change in OD_600_ after being cultured in simulated gastric fluid at pH 2 and 3 for 4 h, and showed a slow growth trend at pH 4. In the simulated intestinal fluid, the AJQ03 strain showed an increasing growth trend, indicating an ability to survive and grow in the intestinal environment.

The development of probiotics relies on the absence of harm to the host (Wang et al., 2019). Therefore, this study evaluated the *in vitro* and *in vivo* safety of strain AJQ03. Hemolysis and drug resistance were used as indicators for *in vitro* safety assessment. Hemolysis, as a virulence factor, can cause symptoms such as anemia in the host, making non-hemolytic strains more suitable for use as probiotics (Nandi et al., 2017). Analysis demonstrated that the AJQ03 strain did not exhibit hemolytic activity on blood plates. Antibiotic resistance genes induced through the overuse of antibiotics can be transmitted, altered, or acquired among bacteria, with serious implications for human health and public safety (Hernando-Amado et al., 2020). Consequently, drug sensitivity is a crucial safety index. Šimunović et al. (2022) tested the antibiotic susceptibility testing of *B. subtilis* PS-216, which showed susceptibility to eight clinically important drugs. Wang J. et al. (2022) found that *B. subtilis* and *B. licheniformis* isolated from the intestinal tract of *Rhynchocypris lagowskii* exhibited good antibiotic sensitivity. Consistent with these findings, our study indicated that strain AJQ03 was sensitive to all 32 antibiotics, showing no resistance.

For safety studies in animal models, the *in vivo* toxicity evaluation guidelines for chemicals proposed by the Organization for Economic Co-operation and Development (OECD) have been meticulously applied in probiotic research (Haranahalli Nataraj et al., 2023). In this study, clinical symptoms, organ indices, intestinal colonization effects, and changes in intestinal flora composition were used as indicators for *in vivo* safety evaluation through both feeding and intraperitoneal injection methods. Jia et al. (2024) reported no apparent organ toxicity in the mice gavaged with *Lactococcus garvieae* HMV18. Similarly, Zhang et al. (2022) found that the acute oral toxicity test of lactic acid bacteria had no adverse effects on organ indices in Kunming mice. In this study, autopsies revealed no distinct pathological changes in the overall appearance or size of internal organs in both groups. Furthermore, no significant differences were observed in organ injury scaling (OIS) between the two groups (*p* > 0.05). These findings indicate no apparent organ toxicity in fish fed or injected with *B. subtilis* AJQ03, consistent with previous reports.

Colonization ability is considered a key screening criterion for selecting potential probiotics, as it enables them to inhibit pathogens by adhering and colonizing intestinal epithelial cells (Vine et al., 2004). Liang et al. (2024) demonstrated that *B. paralicheniformis* successfully colonized the mouse intestine for up to 17 days. Similarly, in this study, on day 14 after instillation, the foregut still harbored 0.69 × 10^2^ CFU/g of strain AJQ03, indicating excellent colonization ability. Intestinal microflora directly influence the health status of an organism, including intestinal function and immunity, and are associated with various diseases (Torrecillas et al., 2023).

Microbial diversity is of pivotal importance to the functioning of the gastrointestinal ecosystem (Hembrom-Preety et al., 2023). Wang M. et al. (2022) found that the *B. subtilis* D1-2-supplemented groups had higher intestinal microbial richness and diversity than the control group, and the results of the present experiment showed that feeding strain AJQ03 resulted in a significant increase in the ACE, Chao, and Simpson indices, which improved species richness as well as uniformity of distribution. Zhang et al. (2014) found that a notable reduction in the Shannon index following the administration of *Bacillus subtilis*, which is in alignment with the findings of the present study. This may be attributed to the fact that *Bacillus subtilis* secretes antimicrobials and produces a probiotic effect by inhibiting the growth of intestinal pathogens (Cutting, 2011). Additionally, *Bacillus* has the ability to colonize the small intestine (Tam et al., 2006), which may facilitate the colonization of specific microbiota. The results of this experimental study indicated that the administration of strain AJQ03 has the potential to regulate and shape the bacterial diversity within the eel gut. However, the precise mechanism of action remains to be fully elucidated.

Dominant intestinal phyla in fish following probiotic feeding include Proteobacteria, Firmicutes, and Bacteroidetes (González-Félix et al., 2018; Gupta et al., 2019). Here, at the phylum level, the gut microbiota was primarily composed of Proteobacteria, Firmicutes, Bacteroidetes, and Actinobacteria, aligning with previous investigations. The dominant phylum in the test group was Firmicutes, while the main dominant phylum in the control group was Proteobacteria. Firmicutes is mainly involved in essential metabolic activities in the intestinal tract, enhancing host immunity, promoting intestinal development, and potentially preventing infection by pathogenic bacteria (Liang et al., 2023). Conversely, an increased abundance of Proteobacteria may lead to structural destabilization of the host gut flora, resulting in metabolic disorders and intestinal inflammation (Shin et al., 2015; Liu et al., 2022). At the genus level, the relative abundance of *Bacillus* in the intestine of the test group increased significantly, indicating that strain AJQ03 successfully colonized the intestinal tract. The relative abundances of *Klebsiella*, *Pseudomonas*, and *Aeromonas* were reduced. *Aeromonas* is known to invade, colonize, and damage host cells, leading to pathogenic activity. Notably, *Aeromonas* is a major pathogen in *A. japonica* culture (Carusi et al., 2024) causing various health issues ranging from gastrointestinal infections and ulcers to hemorrhagic septicemia. Feeding strain AJQ03 effectively reduced the abundance of *Aeromonas*, thereby decreasing disease incidence. From the perspective of intestinal microorganisms, strain AJQ03 exhibits a clear colonizing effect and efficacy in improving the intestinal flora composition, making it a promising candidate for future applications.

## 5 Conclusion

This study selected strain AJQ03 as a potential probiotic candidate based on its strong adhesion capabilities, inhibition of bacterial adhesion, and hydrolase production. Strain AJQ03 exhibited robust tolerance to various pH levels, NaCl concentrations, bile salt concentrations, and simulated gastrointestinal fluids, with no resistance to antibiotics or hemolytic activity. *In vivo* safety tests confirmed that strain AJQ03 had no toxic side effects in fish. Additionally, feeding strain AJQ03 significantly reduced the presence of pathogenic or opportunistic pathogens in the gut, while significantly increasing the number of beneficial bacteria that improve the intestinal environment. Therefore, strain AJQ03 shows great promise for use in aquaculture and provides a solid foundation for the development of fish-derived probiotic formulations.

## Data Availability

The original contributions presented in the study are included in the article/supplementary material, further inquiries can be directed to the corresponding author.
